# Supplementary material for: Cognitive stress changes the attributes of the three heads of the triceps brachii during muscle fatigue
Source: PLoS One. 2020 Jan 30;15(1):e0228089. doi: 10.1371/journal.pone.0228089 (PMC6992167; doi:10.1371/journal.pone.0228089)
Supplement: Attachment — Submitted filename: Response to Reviewers.docx [file pone.0228089.s001.docx]

|  | In the revised discussion the authors added that “spectral parameters are less susceptible to neuro-motor noise than temporal parameters and are thus better approximators of peripheral muscle fatigue at different psychological states.” Please elaborate on how this conclusion can be drawn from the results of the present study since the presented methodology does not measure neural noise (cf. relevant comment of previous review). | We apologize for the misleading statement. The term ‘neuro-motor noise’ is inaccurate. The statement has been replaced by, “spectral parameters are less susceptible to cognitive stress than temporal parameters and are thus better approximators of peripheral muscle fatigue at different psychological states.” | P16,L6-8 |
| --- | --- | --- | --- |
|  | In the present study, the two hypotheses are confirmed while the third is rejected. In the introduction part the third hypothesis is based on the second: “…the presence of CS likely induces a relatively greater decrease in the MPF and MDF and thus a greater ROF than that observed under normal condition”. Please elaborate why, while the second hypothesis is confirmed, the third in rejected, clearly in the discussion part. | We agree that the third hypothesis appears as derivative of the second hypothesis. In view of this, the third hypothesis could be misleading and may not serve any purpose. Hence, we decided to remove the hypothesis. Subsequently, the concerned lines in discussion section has been revised, and reads, “Taken together, it appears that the findings of this study concur with those in literature, suggesting similar behavior of ROF for both isometric and dynamic contractions during CS.” | P15, L1-3 |
|  | Page 14/Line15-17: Please provide a literature reference for the statement that: “At low exercise intensities, the extended time to task failure might cause central fatigue and might thus negatively affect the ET, NR and ROF.” | We agree with the reviewer’s suggestion. The following reference has been found to be pertinent to the concerned issue:   1. A comparison of central aspects of fatigue in submaximal and maximal voluntary contractions, 2008 | P14, L16 |
|  | Page 17/Line 11: Please change the word “but” to “and”, since it is reasonable a reduced ROF to lead to an increase in the ET and NR. | We agree with the reviewer’s suggestion. Changes have been made in the updated manuscript. | P17, L11 |
|  | Page 17/Line 11: Please cite on what the impact of CS was greater under NF conditions. | We apologize for the lack of information in the concerned sentence. The sentence has now been replaced with “For all three heads, larger differences were observed in the normalized RMS between the NCS and CS exercises under NF compared with Fa conditions.” | P17, L11-13 |
