# Supplementary material for: Cognitive stress changes the attributes of the three heads of the triceps brachii during muscle fatigue
Source: PLoS One. 2020 Jan 30;15(1):e0228089. doi: 10.1371/journal.pone.0228089 (PMC6992167; doi:10.1371/journal.pone.0228089)
Supplement: Attachment — Submitted filename: Response to Reviewers.docx [file pone.0228089.s002.docx]

Response to Editor comments

|  | Editor | Response | Page number |
| --- | --- | --- | --- |
|  | Please have the entire manuscript edited for English expression by a native speaker of English who has knowledge of your field. Language imprudent is required; avoid the first-person writing style. | The updated manuscript has been proofread. The language used in the manuscript is English (United States). | Entire manuscript |
| 1. | One important key word “Muscle” is missing in the title, therefore I would recommend putting this word either with triceps brachii “....triceps brachii muscle...” or just before the word fatigue, “…..triceps brachii during Muscle fatigue” | We thank the editor for pointing out this gap. We propose a new title, “Cognitive stress changes the attributes of the three heads of the triceps brachii during muscle fatigue”. | Title |
| 2. | Define the acronym(s), first time that they are used; such as in Abstract, 1RM, RMS, MPF and MDF. Please check the entire manuscript. | The acronyms have been defined at the first place of use, as well as in the abstract. | Entire manuscript |
| 3. | The keywords need to arrange based on alphabetical order. | The keywords have been arranged in alphabetical order. | Keywords |
| 4. | In the Introduction, first paragraph: only two references (one is very old), are not enough to support the statement. Specially the external factor “Temperature”,. Please add relevant and recent articles. In the same paragraph, instead of writing “[1] revealed that…”, please put the author name(s). Similarly, please add reference(s) to support your first two statements in the second paragraph. | For temperature, the classic study by Michael Holewijn has been added. Further, review article by Enoka has also been added to support the first paragraph of the manuscript.  References have also been added for the first two lines of the second paragraph. | First two paragraphs of introduction |
| 5. | In the Introduction, “The triceps brachii, as the largest arm muscle…’, is there any right reference other than your own article to support this? Because, Biceps Brachii muscle is recognized as largest and right place to place EMG electrode as per SENIAM. | We apologies for the misleading statement, the new statement reads as, “The triceps brachii, which is only muscle in the posterior compartment of the arm, acts as an elbow extensor, a horizontal arm abductor, and an antagonist in elbow flexion.” | P4, L5-6 |
| 6. | Please revise the sentence “Because the TB is close to the CNS..”, because, facial muscle, eye muscle etc. are the close to CNS. | We do agree that the statement could be misleading. The new statement reads, “The anatomical distance of the TB from the CNS renders it more susceptible to the effects of cognitive stress when compared to distal skeletal muscles such as the forearm and leg muscles.” | P4, L7-8 |

Response to Reviewer comments

|  | Reviewer Comments | Response | Page number |
| --- | --- | --- | --- |
|  | However, I still have some concerns, especially on the fact on how the results are related to neuro-motor noise. When conclusions for motor units behavior are drawn from bipolar surface EMG, this should be done very carefully. The conclusion that the greater RMS increase during the CS exercise compared to the NCS could be due to neuro-motor noise, still needs justification. It is possible that an increase in EMG activity, can be explained due to the increased antagonist-agonist co-contraction, which can be an indication of higher neuro-motor noise in order to counteract the kinematic variability [as stated by “The effects of workplace stressors on muscle activity in the neck shoulder and forearm muscles during computer work: a systematic review and meta-analysis”], but there are many other factors as well. For example, it is possible that the greater duration of the exercise under CS may explain the greater RMS increase. Furthermore, the authors have not investigated neither co-contraction nor kinematics variability, which could be indicators for motor noise existence, so that they can argue that motor noise is the factor explaining RMS increase. | We do agree with the reviewer that change in RMS values could be due to many factors and the results of the investigated parameters are not related to neuro-motor noise.  We postulated that the if cognitive stress is induced in the subject, it may cause increase in the neuro-motor noise (as suggested by literature) and this increased neuro-motor noise may cause kinematic variability and agonist-antagonist co-contraction. Our protocol ensures that CS is induced in the subject, and hence we expect the outcomes of this phenomenon.  Nevertheless, these phenomenon (kinematic variability and agonist-antagonist co-contraction) were not analyzed in the current study, hence we revised the discussion section accordingly. We have omitted the parts of the manuscript that discusses the neuro-motor noise as sole reason of increased RMS. | Response |
|  | Specific Comments |  |  |
| 1. | In the previous review I commented that a figure depicting the experimental procedure would be helpful, meaning the temporal set up, e.g. first warming up then 2 min rest, 3 RM (the between intervals), etc. I believe that the words “experimental set up” confused the authors, and added a figure depicting the spatial set up of the experiment. Nevertheless, this is also described in figure 1, so a suggestion could be to keep either figure 1 or figure 2 and add another figure depicting the temporal experimental procedure. | Figure 2 has been removed and a new figure has been added in the updated manuscript describing the temporal experimental procedure. | Figure 2 |
| 2. | Page 7/Line 16-18: At the present study EMG parameters (RMS, MDF and MPF) were compared between different conditions. In order to investigate the effect of the different conditions, motion should be the same, i.e. constant ROM at constant velocity, since these two parameters influence the EMG signal. Thus, I disagree with the statement that the present study did not require the joint angles to be measured or controlled during sEMG data acquisition as data was required throughout the entire ROM. | We found this statement misleading and the same information has been given at other places (data analysis), hence decided to remove it.  We agree with the reviewer that the sEMG signals obtained at different joint angles could be different, and speed and ROM significantly affect the sEMG signals.  We do agree that our statement could be misleading. By different joint angles, we actually mean, “obtaining sEMG signals at particular elbow joint angle in every cycle instantaneously”. The focus of the study did not require sEMG to be instantaneously sampled at particular joint angles, but rather the protocol required speed of motion to be monitored and controlled throughout ROM and it was done using a custom-made LabVIEW program. The ROM and all relevant appropriate joint angles were monitored by a dedicated assistant, who ensured the starting point, full elbow extension and back to starting point for forearms. | P9, L8; Data Analysis |
| 3. | Page 9/Line 8-10: I have some concerns on how accurate the duration of one repetition can be defined from EMG signal. There is an activation of TB during elbow flexion (eccentric phase) and also during elbow extension (concentric phase) with a small silent window at the end of each repetition. The duration of this silent time window could be fatigue depended. | The algorithm, responsible for the measurement of repetition duration, identify continuous, non-overlapping segments of sEMG signals. The silent window was part of the repetition duration. Each repetition duration includes the active phase and the silent window.  While we agree that the duration of the silent window is fatigue dependent, however, it was ensured during the experiment that subject should not pause at any instant throughout the exercise duration. If a subject pause during the exercise, the data was discarded, and experiment was reperformed. | P9, L4-6 |
| 4. | Results session continuous to be complicatedly demonstrated. For example, it is mentioned that “Under the NF condition, all three heads were significantly different (p<0.05) between the NCS and CS exercises for RMS, while only the long head was statistically significant for MPF and MDF.” But there is no information on which variable is higher. Furthermore, in figure 5, 6 and 7 the statistically significant differences are not indicated. | The result section has been re-written as per the recommendations of the reviewer. | Result section |
| 5. | It would be easier for the reader to follow and understand the main findings of the study, if at the beginning of the discussion the main findings are described and whether these findings confirm or reject the hypotheses (a. that the RMS activity of the lateral and long heads would increase in the presence of cognitive stress because these are larger muscles, whereas that of medial head would remain and b. that the values of MPF and MDF for the lateral and long heads would experience a relatively higher decrease in the presence of cognitive stress compared with those for the medial head). Furthermore, at the introduction is stated that the rate of fatigue (ROF) is expected to be higher in the presence of cognitive stress than under normal conditions. However, this expectation was not confirmed from the results. A restructure of the discussion where the justification, of the confirmation or rejection of the hypotheses should be done in order to improve discussion clarity. | The discussion section has been restructured and new information has been added. Further, the hypothesis has been revised for better clarity. | Discussion section |
| 6. | There is a significant interaction found between mental load and fatigue development in the RMS and in all the heads of TB for RMS and MPF, which is not discussed in the discussion part. | The interaction results are now discussed in the discussion section. | P16, L3-8 |
