# Supplementary material for: Cognitive stress changes the attributes of the three heads of the triceps brachii during muscle fatigue
Source: PLoS One. 2020 Jan 30;15(1):e0228089. doi: 10.1371/journal.pone.0228089 (PMC6992167; doi:10.1371/journal.pone.0228089)
Supplement: Attachment — Submitted filename: Response to reviewers.docx [file pone.0228089.s004.docx]

|  | Comments | Response |  |
| --- | --- | --- | --- |
| 1 | In the introduction part the second hypothesis, i.e. the presence of CS would induce a relatively greater decrease in the MPF and MDF is based on the assumption that there would be greater muscle activity during exercise. Indeed, the authors found greater RMS values under CS. Interestingly the ROF is lower and the duration is greater under CS, albeit the higher energy cost during task execution under CS, as indicated by the higher EMG activity. Is it possible that the differences in task duration may have influenced the MPF and MDF reduction between the different conditions (CS vs NCS)? This issue should be explained/commented in the discussion section of the paper. | Though lower ROF, greater differences in MPF and MDF between NF and Fa were observed during CS exercise, which could be due to increased ET during CS. This particular point has already been discussed in the discussion section on P15, L20-22. The reviewer has pointed out another effect that might have influenced MPF and MDF. We do agree with the reviewer over this and P15, L22-25 discussed this matter. The lines reads, “It is interesting to note that although the drop in MPF and MDF is higher under CS, their rates of drop (ROF) was found lower, albeit the higher energy cost during task execution under CS, as indicated by the higher sEMG activity. The reason for this could be the increased ET observed during the CS exercise.” | P15, L20-23 |
| 2 | Page 14/ Line 23-25: The authors state that “The higher ET observed in our study might also be responsible for the lower ROF calculated during exercise with CS”. As the higher ET is the result of the art that the neuromuscular system is operated and not vice versa, it is more reasonable to write that the lower ROF observed in the study might be responsible for the higher ET observed during exercise with CS. Please revise the discussion accordingly. | We agree with the reviewer and the stated sentence has been rephrased as, “The lower ROF observed in this study might be responsible for the higher ET during the CS exercise”. | P14, L23-24 |
